# Evaluation of Glycosylated FlpA and SodB as Subunit Vaccines Against *Campylobacter jejuni* Colonisation in Chickens

**DOI:** 10.3390/vaccines8030520

**Published:** 2020-09-11

**Authors:** Prerna Vohra, Cosmin Chintoan-Uta, Vanessa S. Terra, Abi Bremner, Jon Cuccui, Brendan W. Wren, Lonneke Vervelde, Mark P. Stevens

**Affiliations:** 1 The Roslin Institute and Royal (Dick) School of Veterinary Studies, University of Edinburgh, Edinburgh EH25 9RG, UK; Cosmin.Chintoan-Uta@roslin.ed.ac.uk (C.C.-U.); Abi.Bremner@roslin.ed.ac.uk (A.B.); Lonneke.Vervelde@roslin.ed.ac.uk (L.V.); Mark.Stevens@roslin.ed.ac.uk (M.P.S.); 2 Institute for Immunology and Infection Research, School of Biological Sciences, Charlotte Auerbach Road, University of Edinburgh, Edinburgh EH9 3FL, UK; 3 Faculty of Infectious and Tropical Diseases, London School of Hygiene and Tropical Medicine, London WC1E 7HT, UK; Vanessa.Terra@lshtm.ac.uk (V.S.T.); Jon.Cuccui@lshtm.ac.uk (J.C.); Brendan.Wren@lshtm.ac.uk (B.W.W.)

**Keywords:** glycoconjugate vaccines, *Campylobacter*, chickens

## Abstract

*Campylobacter jejuni* is the leading bacterial cause of human gastroenteritis worldwide and the handling or consumption of contaminated poultry meat is the key source of infection. *C. jejuni* proteins FlpA and SodB and glycoconjugates containing the *C. jejuni N*-glycan have been separately reported to be partially protective vaccines in chickens. In this study, two novel glycoproteins generated by protein glycan coupling technology—G-FlpA and G-SodB (with two and three *N*-glycosylation sites, respectively)—were evaluated for efficacy against intestinal colonisation of chickens by *C. jejuni* strain M1 relative to their unglycosylated variants. Two independent trials of the same design were performed with either a high challenge dose of 10^7^ colony-forming units (CFU) or a minimum challenge dose of 10^2^ CFU of *C. jejuni* M1. While antigen-specific serum IgY was detected in both trials, no reduction in caecal colonisation by *C. jejuni* M1 was observed and glycosylation of vaccine antigens had no effect on the outcome. Our data highlight inconsistencies in the outcome of *C. jejuni* vaccination trials that may reflect antigen-, challenge strain-, vaccine administration-, adjuvant- and chicken line-specific differences from previously published studies. Refinement of glycoconjugate vaccines by increasing glycosylation levels or using highly immunogenic protein carriers could improve their efficacy.

## 1. Introduction

*Campylobacter* is the most common bacterial cause of human gastroenteritis globally and is estimated to have caused 95 million illnesses and 21,000 deaths worldwide in 2010 [1]. Source attribution studies unequivocally implicate the consumption and handling of contaminated poultry meat as a key risk factor for human infections [2], and it is estimated that up to 80% of human cases may be linked to the avian reservoir [3]. In the United Kingdom, 63,946 laboratory-confirmed cases of human campylobacteriosis were recorded in 2017 [4], with 9.3 cases predicted to be unreported for every one captured by national surveillance [5] and an estimated annual cost to the economy of GBP 50 million [6]. Surveys of fresh retail chicken across the United Kingdom during the period 2017–2018 found 56% to be contaminated with *Campylobacter* [7].

Campylobacteriosis in humans ranges in severity from mild gastroenteritis to acute self-limiting haemorrhagic diarrhoea involving severe inflammation and may lead to long-term sequelae including reactive arthritis and inflammatory neuropathies such as Guillain–Barré Syndrome [8,9,10,11]. Poultry are not generally affected by *C. jejuni* despite carrying large numbers of bacteria in their gastrointestinal tract [12,13]. However, some studies have reported a decrease in growth performance in chickens harbouring *C. jejuni* asymptomatically that may be associated with physiological changes in the intestines [14]. Moreover, in some broiler breeds *C. jejuni* has been associated with clinical signs such as gut damage, inflammatory responses and diarrhoea [15]. Control of *Campylobacter* in poultry may, therefore, enhance productivity and welfare in some instances in addition to reducing a key foodborne zoonosis. It has been predicted that a 100-fold reduction in *C. jejuni* on chicken carcasses could reduce human infections by 30-fold [16].

Vaccination is one strategy being developed to control *C. jejuni* in poultry and several conserved and immunodominant *C. jejuni* protein antigens have been explored as candidates either as subunit vaccines or vectored in live-attenuated *Salmonella*. These include flagellum-related antigens such as FlaA, FliD and FspA [17,18,19], membrane transport proteins such as CjaA and CjaD [20,21,22,23,24], surface-exposed proteins such as Peb1A, CmeC, CadF and FlpA [18,21], and the superoxide dismutase SodB [25]. The success of these vaccines, tested using different schedules and *C. jejuni* strains, has been variable, with reductions in intestinal *C. jejuni* loads from 1.5 to 6 log_10_ being reported. *C. jejuni* carbohydrates have also been evaluated as vaccines, including the capsular polysaccharide [26,27,28] and *N*-linked glycans [29,30,31]. Reductions in *C. jejuni* colonisation from 4 to 6 log_10_ have been reported in chickens vaccinated with an inactive form of *Corynebacterium diphtheriae* ToxC conjugated to the heptasaccharide [28]. Further, oral vaccination of chickens with *E. coli* decorated with the *C. jejuni N*-glycan was associated with a reduction in *Campylobacter* colonisation of up to 10 log_10_ [29], and vaccination with outer membrane vesicles of *N*-glycan decorated *E. coli* have been reported to reduce *C. jejuni* colonisation by almost 4 log_10_ [30]. Thus, coupling effective *C. jejuni* protein antigens with the *C. jejuni N*-glycan could be a viable strategy to enhance the efficacy of *Campylobacter* vaccines.

The naturally occurring *N*-glycosylation system of *C. jejuni*, encoded by the *pgl* locus, is responsible for the production of the assembled heptasaccharide consisting of GalNAc-GalNAc-(Glc)-GalNAc-GalNAc-GalNAc-diNAcBac (where diNAcBac is 2,4-di-acetamido-2,4,6-trideoxy-D-glucose) on an undecaprenyl-pyrophosphate (Und-PP) carrier. This glycan is highly conserved in *Campylobacter* species and is transferred by the enzyme PglB onto asparagine residues within acceptor proteins containing the D/E-Y-N-X-S/T glycosylation motif [32]. The *pgl* locus is constitutively expressed and is responsible for the *N*-glycosylation of numerous periplasmic and membrane proteins and plays an important role in the colonisation of chickens and virulence in humans [33,34,35,36,37]. The transfer of heterologous loci for polysaccharide biosynthesis and *C*. *jejuni pglB* into *E. coli* has enabled the production of recombinant glycoconjugate proteins in a low-cost and effective process termed protein glycan coupling technology (PGCT) [38,39]. PGCT enables the production of novel glycoproteins owing to the ability of PglB to transfer any sugar moiety assembled on an Und-PP carrier onto an acceptor protein containing the D/E-Y-N-X-S/T glycosylation motif. It has been used to produce novel vaccines against *Francisella tularensis* [40], *Staphylococcus aureus* [41], *Shigella flexneri* [42] and *Streptococci* [43] by the addition of O-antigen or capsular antigens of these pathogens onto carrier proteins. In this study, we used PGCT to couple the *C. jejuni* heptasaccharide, which has been reported to be protective against *Campylobacter*, to the *C. jejuni* antigens FlpA and SodB, which have previously been shown to reduce *C. jejuni* colonisation in chickens by 3 and 1.5 log_10_, respectively [18,25]. We evaluated the efficacy of these novel glycoconjugates relative to unglycosylated variants as subunit vaccines against experimental *C. jejuni* challenge with strain M1 at two doses in chickens and discuss the variables that need to be considered in poultry vaccination studies with *C. jejuni.*

## 2. Materials and Methods

### 2.1. Bacterial Strains and Culture Conditions

*Campylobacter jejuni* M1 [44] was routinely cultured on charcoal–cephoperazone–deoxycholate agar (CCDA) at 40 °C under microaerophilic conditions (5% O_2_, 5% CO_2_ and 90% N_2_). Liquid cultures were prepared in Mueller–Hinton (MH) broth that was allowed to equilibrate with the microaerophilic atmosphere overnight before inoculation and incubation for 16 h with shaking at 400 rpm. For oral challenge of chickens, liquid cultures were adjusted based on a standard curve of CFU mL^−1^ relative to absorbance at 600 nm, with serial dilution where required, to obtain the desired challenge dose. Inocula used in chicken studies were confirmed by retrospective plating of 10-fold serial dilutions on CCDA and determination of viable counts after incubation for 48 h.

### 2.2. Animal Experiments with Layer Chickens

White Leghorn chickens from *Campylobacter*-free flocks were obtained on the day of hatching from a Home Office licensed breeding establishment and housed in groups of up to 30 in colony cages. Groups were of mixed sex and individuals were wing-tagged for identification. Water and sterile irradiated feed based on vegetable protein (DBM Ltd., Broxburn, UK) were provided ad libitum. Animal experiments were conducted at the Moredun Research Institute according to the requirements of the Animals (Scientific Procedures) Act 1986 under project licence PCD70CB48, with the approval of the local Ethical Review Committee. Chickens were monitored twice daily. Post-mortem examinations were conducted following culling by cervical dislocation.

### 2.3. Dose Titration and Colonisation Dynamics of C. jejuni M1

To determine the minimum challenge dose of *C. jejuni* M1 required for reliable intestinal colonisation of chickens, an overnight culture containing approximately 10^9^ CFU ml^−1^ was diluted in MH broth to prepare four inocula such that a dose of 100 μL would contain approximately 10, 10^2^, 10^3^ and 10^4^ CFU of *C. jejuni* M1, respectively. At day 15 of life, six chickens per group were challenged by oral gavage with 100 μL of these cultures. Post-mortem examinations were performed 7 days post-infection. Contents from both caeca of each bird were mixed together in equal quantities and 10-fold serial dilutions were prepared in phosphate-buffered saline (PBS) and plated on CCDA to determine viable *C. jejuni* per gram of caecal contents in individual chickens.

Once the minimum dose of *C. jejuni* M1 that reliably colonised all chickens in a group was defined, nine chickens were challenged with the minimum dose at day 15 of life. Post-mortem examinations of three chickens were conducted on days 1, 3 and 5 post-infection, respectively. Bacteria were enumerated from both caeca of each bird by plating 10-fold serial dilutions on CCDA as above to determine the dynamics of *C. jejuni* M1 colonisation.

### 2.4. Preparation of FlpA, SodB and Their Glycosylated Variants

Briefly, full length FlpA and SodB from *C. jejuni* M1 were modified genetically to enable *N*-glycosylation by PglB by the addition of one *N*-terminal and one *C*- terminal DQNAT glycosylation site. SodB was further modified to contain a third glycosylation site at position 96–100 by a single amino acid substitution (Q100T). The proteins contain a signal peptide (PelB) to direct them to the periplasm for glycosylation and a C-terminal 6-His tag for affinity purification (Figure S1). The constructs were cloned into an isopropyl β-D-1-thiogalactopyranoside (IPTG)-inducible plasmid, pEXT20, thus generating plasmids pFlpA and pSodB (*amp^R^*). The plasmids were introduced by electroporation into *E. coli* SDB1, a *wecA-waaL*-strain suitable for PGCT, containing either pACYC*pgl* (*cat^R^*), a plasmid that contains all the necessary genes to produce the *C. jejuni N*-glycan and a functional PglB to mediate *N*-glycosylation [39] or pACYC*pgl*::*pglB*KO (*cat^R^ kan^R^*), where the PglB is non-functional. This was done to ensure that protein preparation methods were comparable for both the glycosylated and unglycosylated variants and that any background contaminants would be common to both preparations. Transformants selected on LB agar supplemented with 100 µg ml^−1^ ampicillin and 100 µg mL^−1^ chloramphenicol produced glycosylated FlpA (G-FlpA) and SodB (G-SodB) whereas those transformed with pACYC*pgl*::*pglB*KO (*cat^R^ kan^R^*) and selected on LB agar supplemented with 100 µg mL^−1^ ampicillin and 50 µg mL^−1^ kanamycin produced unglycosylated FlpA and SodB.

For vaccine production, transformants were grown overnight at 37 °C on LB broth with suitable antibiotics. The following day, the cultures were diluted 1:100 and grown at 37 °C under shaking conditions until an OD_600_ of 0.5 was reached. A total of 1 mM IPTG was added to the cultures and they were grown for a further 16 h at 37 °C. Cell pellets were collected by centrifugation at 5300× *g* for 30 min at 4 °C, resuspended in ice-cold lysis buffer (50 mM NaH_2_PO_4_, 300 mM NaCl, 10 mM imidazole, pH 8.0) and subjected to five rounds of mechanical lysis using a pre-chilled Stansted High Pressure Cell Disruptor (Stansted Fluid Power Ltd., Harlow, UK) under 60,000 psi (410  MPa) in continuous mode. The lysate was centrifuged at 10,000× *g* for 60 min at 4 °C, and the supernatant was collected for protein purification using Ni-affinity chromatography. The supernatant was combined with Ni-NTA resin (Ni-NTA, Qiagen, Germany) for 1 hour at 4 °C. The column was then washed with 200 mL of wash buffer (50 mM NaH_2_PO_4_, 300 mM NaCl, 20 mM imidazole, pH 8.0) and the proteins were eluted using 2 mL of elution buffer (50 mM NaH_2_PO_4_, 300 mM NaCl, 250 mM imidazole, pH 8.0). Protein fractions were pooled and concentrated using buffer exchange columns Vivaspin 2 (Vivaproducts, Littleton, USA) into PBS. The purity of glycoconjugates and antigens was assessed by Coomassie staining of proteins resolved by sodium dodecyl sulphate-polyacrylamide gel electrophoresis (SDS-PAGE) and western blotting using anti-His (Clone J099B12, BioLegend, San Diego, USA) at 1:1000 followed by anti-mouse IgG (H+L, Dylight 680, Cell Signaling Technology) at 1:10,000 for protein detection and soybean agglutinin (Vector Laboratories, Burlingame, USA) at 1:4000, followed by IRDye Streptavidin (LiCOR) at 1:3000 dilution for glycan detection. Protein concentration was determined by NanoDrop (ThermoFisher, Waltham, USA), using the extinction coefficient determined for the modified proteins calculated using ProtParam (Expasy): FlpA extinction coefficient 54,780 M^−1^ cm^−1,^ Abs (0.1%) = 1.117, Molecular weight (MW) = 49042.09 Da, and SodB extinction coefficient 47,120 M^−1^cm^−1,^ Abs (0.1%) = 1.623, Molecular weight (MW) = 28878.43 Da. Quantification of both glycosylated and unglycosylated antigens for vaccination was based on total amount of protein.

### 2.5. Trials to Evaluate the Efficacy of FlpA, SodB and Their Glycosylated Variants as C. jejuni Vaccines

Vaccines were prepared by mixing purified glycosylated or unglycosylated proteins with Montanide^TM^ ISA 70 VG (Seppic, Rungis, France) at a ratio of 30% protein and 70% adjuvant. Groups of 30 chickens were vaccinated on days 6 and 16 of life intramuscularly with 100 µL protein divided equally between two pectoral muscles, containing 240 µg of FlpA and G-FlpA or 138 µg of SodB and G-SodB. Mock-vaccinated chickens were injected with a mixture of 30% PBS and 70% Montanide^TM^ ISA 70 VG. Two independent trials of this design were performed. At day 20 of life, 4 days after secondary vaccination, birds were challenged by oral gavage with 100 µl of culture containing either a dose of 10^7^ CFU of *C. jejuni* M1 (high dose challenge trial) or 10^2^ CFU of *C. jejuni* M1 (minimum dose challenge trial). In both trials, post-mortem examinations were performed on days 7, 17 and 28 post-infection. The contents from both caeca of each bird were collected as described above and 10-fold serial dilutions prepared in PBS and plated on CCDA to determine viable counts per gram. Blood was collected by cardiac puncture at post-mortem examination and serum was stored at −80 °C following the centrifugation of clotted blood at 1000× *g* for 10 min at 4 °C.

### 2.6. Analysis of Humoral Immune Responses Following Vaccination

Antigen-specific serum IgY levels were quantified by enzyme-linked immunosorbent assays (ELISA). Briefly, 96-well plates were coated with 0.5 μg mL^−1^ FlpA, G-FlpA, SodB or G-SodB in carbonate–bicarbonate buffer and incubated at 4 °C overnight. Plates were washed with PBS containing 0.005% (*v*/*v*) Tween-20. Serum samples were diluted in PBS and 100 μL of diluted serum was added per well. Serum dilutions of 1:500 for FlpA and G-FlpA and 1:300 for SodB and G-SodB were selected based on the output from chequer-board assays to identify dilutions that would produce absorbance readings in the linear range. Serum was tested against the glycosylated vaccine antigen to quantify vaccine-specific responses and against the cognate unglycosylated antigen to determine whether higher responses were observed against the glycosylated antigens. Control wells were used, to which no serum was added. To confirm the specificity of the IgY detected, serum from five chickens vaccinated with FlpA or G-FlpA were tested against SodB and G-SodB and vice versa. Plates were incubated at 37 °C for 1 h and then washed as above. Rabbit anti-chicken IgY-horseradish peroxidase (HRP) at 1:3000 (Sigma, UK) was used to detect bound serum IgY. Plates were washed twice. Tetramethylbenzidine (TMB) substrate (BioLegend, UK) was then added and the plates were incubated for 10 min at room temperature in the dark. The reaction was stopped using 2 M H_2_SO_4_ and absorbance at 450 nm adjusted against absorbance at 620 nm (*A*_450/620_) was measured using a plate reader with background correction using the values of the control wells (Multiskan Ascent, ThermoFisher, Waltham, USA).

### 2.7. Statistical Analysis

Statistical tests were performed in GraphPad Prism version 8.00 (GraphPad Software, San Diego, USA). Differences in colonisation levels and humoral responses between groups of chickens in the vaccination trials at each time point were analysed using the Kruskal–Wallis test followed by Dunn’s multiple comparison test. *P* values of ≤0.05 were considered to be statistically significant. Data are represented graphically as median values with 95% confidence intervals.

## 3. Results

### 3.1. Determining the Minimum Dose of C. jejuni M1 Required for Reliable Intestinal Colonisation

**Figure 1:**
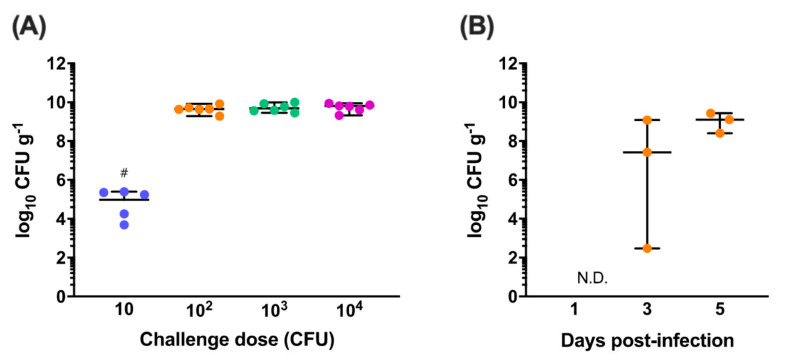
Dose titration and colonisation dynamics of *C. jejuni* M1. (**A**) White Leghorn chickens were orally challenged with doses of *C. jejuni* M1 ranging from 10 to 10^4^ colony-forming units (CFU) and caecal colonisation was determined at 7 days post-infection. In the group challenged with 10 CFU (●), five of the six chickens were colonised at a median level of 4.9 log_10_ CFU g^−1^. *C. jejuni* could not be detected in one chick in this group (#). All chickens challenged with 10^2^ CFU (●), 10^3^ CFU (●) and 10^4^ CFU (●) were colonised at a median level of over 9.6 log_10_ CFU g^−1^. A dose of 10^2^ CFU was defined as the minimum dose for reliable colonisation. (**B**) The caecal colonisation dynamics of *C. jejuni* M1 were determined following a challenge with 10^2^ CFU (●). It was observed that bacterial load increased exponentially over time, reaching a near-maximum median load of 9.1 log_10_ CFU g^−1^.

*C. jejuni* M1 is a human isolate that reliably colonises the gastrointestinal tract of chickens when administered at a dose of 10^7^ CFU [19,21,25]. At lower doses, colonisation and transmission dynamics of isogenic signature-tagged variants of M1 can be unstable and unpredictable in chickens during co-infections [45,46]. Dose titration experiments were performed to determine the minimum challenge dose of *C. jejuni* M1 required for reliable colonisation of the caeca, a key site of persistence of *Campylobacter* in chickens. At 7 days post-infection, the caeca of five of the six chickens challenged with 10 CFU were colonised by *C. jejuni* M1 at a median level of 4.9 log_10_ CFU g^−1^ (Figure 1A). In contrast, all six chickens in the groups challenged with 10^2^, 10^3^ and 10^4^ CFU were colonised at a median level of 9.6, 9.7 and 9.8 log_10_ CFU g^−1^, respectively, as previously observed following high dose challenge with 10^7^ CFU [19,21,25]. A dose of 10^2^ CFU *C. jejuni* M1 was therefore selected as the minimum dose required for reliable colonisation based on these results.

To define the colonisation dynamics of *C. jejuni* M1 following the challenge of chickens with 10^2^ CFU, caecal colonisation levels were determined at days 1, 3 and 5 post-infection (Figure 1B). *C. jejuni* M1 could not be detected in the caeca of infected chickens at day 1 and on day 3 colonisation levels in the chickens ranged from 2.5 to 9.1 log_10_ CFU g^−1^. However, by day 5, all chickens were colonised at a median level of 9.1 log_10_ CFU g^−1^.

### 3.2. Effect of Vaccination on C. jejuni Colonisation

**Figure 2:**
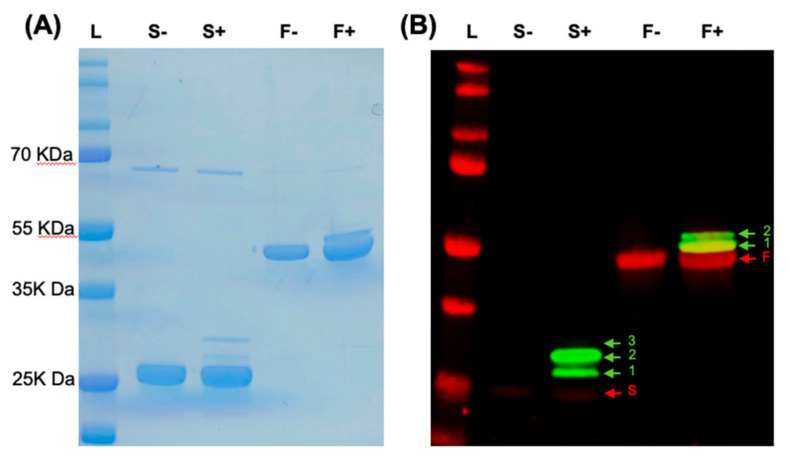
Novel glycoconjugate vaccines against *C. jejuni*. (**A**) SDS-PAGE was used to confirm the size of 6-His-purified FlpA (F) and SodB (S) produced in the presence of pACYC*pgl*::*pglB*KO (−) and their novel glycosylated variants produced in the presence of pACYC*pgl* (+). (**B**) Western blotting was used to confirm the glycosylation status of the vaccines. Anti-His staining in red confirmed the presence of His-tagged antigens of the correct size while anti-glycan staining in green using anti-soybean agglutinin confirmed that the antigens were glycosylated in the presence of an active PglB on pACYC*pgl*. As expected, the addition of *N*-glycans at the available glycosylation sites in G-FlpA and G-SodB resulted in a change in mass of the proteins indicated by the ladder pattern (green arrows with numbers to signify the number of glycans added) observed above the unglycosylated protein (red arrows).

**Figure 3:**
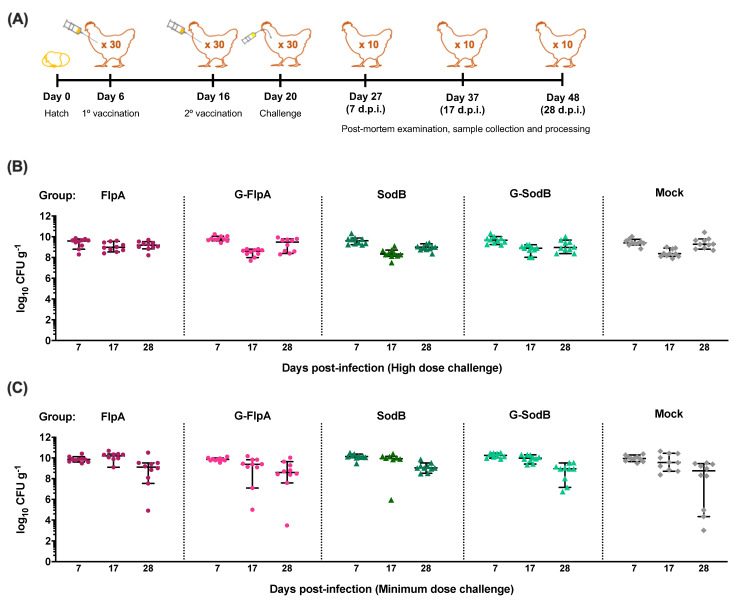
Caecal colonisation by *C. jejuni* M1 post-vaccination and oral challenge. (**A**) White Leghorn chickens were vaccinated with FlpA (●), G-FlpA (●), SodB (▲), G-SodB (▲) or PBS (mock-vaccinated, ♦) mixed with adjuvant at days 6 and 16 of life followed by oral challenge with *C. jejuni* M1 at day 20 of life. Post-mortem examinations were conducted at days 7, 17 and 28 post-infection to study caecal colonisation levels of *C. jejuni* M1 and immune responses. (**B**) Following a high dose challenge of 10^7^ CFU of *C. jejuni* M1, high caecal loads were observed in all groups that were not significantly different between vaccinated and mock-vaccinated groups. (**C**) Similar results were observed in a minimum dose challenge trial in which chickens were challenged with 10^2^ CFU of *C. jejuni* M1, further demonstrating that the challenge dose did not affect the outcome of vaccination.

His-tagged FlpA and SodB and their glycosylated variants (G-FlpA and G-SodB) were affinity-purified from *E. coli* and verified by SDS-PAGE and western blotting to contain proteins of the correct molecular weight modified with up to two or three *C. jejuni N*-glycans in the case of G-FlpA and G-SodB, respectively (Figure 2). However, preparations of G-FlpA and G-SodB were also found to contain large amounts of unglycosylated proteins. Chickens were vaccinated with purified proteins or glycoproteins using the schedule previously described to be protective using FlpA as a vaccine against *C. jejuni* in chickens [18] (Figure 3A). Caecal colonisation levels in vaccinated and mock-vaccinated chickens were determined at days 7, 10 and 28 post-infection in two independent trials of the same design with either 10^7^ CFU *C. jejuni* M1 (Figure 3B) or 10^2^ CFU (Figure 3C). No significant reduction in caecal colonisation by *C. jejuni* M1 was observed in either vaccination trial. In the minimum dose challenge trial, a slight but non-significant reduction in caecal colonisation was observed at day 28 post-infection in all groups compared to colonisation levels at previous time points.

### 3.3. Immune Responses to Vaccination

**Figure 4:**
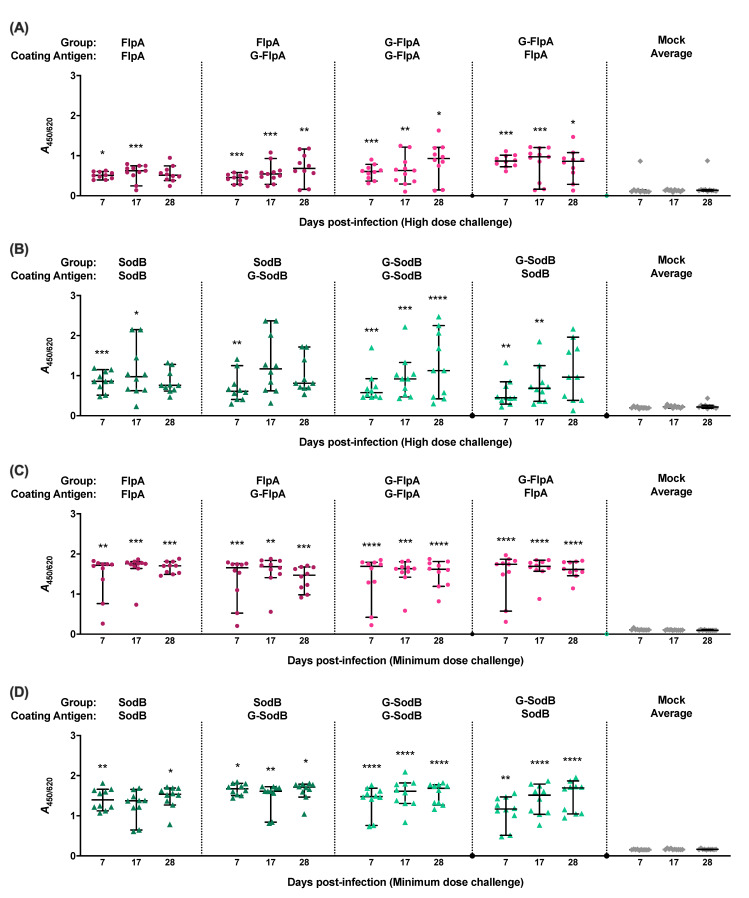
Induction of humoral immune responses. Levels of serum IgY against the administered vaccine antigens were determined in chickens vaccinated with FlpA (●), G-FlpA (●), SodB (▲), G-SodB (▲) or PBS (mock-vaccinated, ♦) at all the time points post-infection. High levels of antigen-specific IgY were found in all vaccinated birds groups compared to mock-vaccinated chickens. This was observed in both the high dose challenge trial (**A**,**B**) as well as the minimum dose challenge (**C**,**D**), although antibody levels were higher in the latter trial. Antibody levels did not have an impact on caecal colonisation levels. Statistically significant *P* values of ≤0.05 compared to the mock-vaccinated group are indicated by asterisks. No differences in serum IgY levels were found between groups vaccinated with glycosylated and unglycosylated vaccine antigens.

Antigen-specific IgY in sera of chickens was quantified by ELISA using the cognate glycosylated or unglycosylated forms of the protein as the capture antigen. Significantly elevated antigen-specific IgY levels were detected in all vaccinated chickens in both trials involving challenge with 10^7^ CFU (Figure 4A,B) and 10^2^ CFU of *C. jejuni* M1 (Figure 4C,D) when compared to the mock-vaccinated chickens. Where birds were vaccinated with G-FlpA or G-SodB, antigen-specific IgY responses were not significantly different when the glycosylated proteins were used as capture antigen compared to when the cognate unglycosylated forms were used. Minimal cross-reactivity was observed when serum from FlpA and G-FlpA vaccinated chickens was tested against captured SodB and G-SodB and vice versa (Figure S2), indicating that the IgY responses measured can be attributed to the specific protein antigens used and not co-purified contaminants from *E. coli*.

## 4. Discussion

Vaccination can be an effective way to control zoonotic bacterial pathogens in poultry, as evidenced by the reduction in human non-typhoidal salmonellosis and egg and meat contamination following the implementation of *S. enterica* serovar Enteritidis and Typhimurium vaccines in laying hens and broiler breeders in many countries. To be effective for poultry, a *C. jejuni* vaccine must reduce intestinal colonisation and provide protection within five to six weeks of life owing to the short lifespan of fast-growing commercial broilers. Protein antigens like FlpA and SodB have been reported to reduce *C. jejuni* colonisation [18,25], as has vaccination with the *C. jejuni N*-glycan coupled to a protein carrier or expressed on the surface of *E. coli* or their outer membrane vesicles [29,30]. In this study, we evaluated if conjugating the *C. jejuni N*-glycan to FlpA and SodB could enhance the protective effect of the novel glycoconjugate vaccines produced.

Traditionally, the production of glycoconjugate vaccines has involved the chemical conjugation of highly-purified polysaccharides and proteins, which is time- and labour-intensive and often produces low vaccine yields, making them relatively expensive. Veterinary vaccines must be cheap to produce and administer on the scale of commercial poultry production. The use of PGCT overcomes the limitations of conventional glycoprotein production by enabling the production of large amounts of glycoproteins in *E. coli* carrying the *C. jejuni pgl* locus. In this study, we used PGCT to generate the novel glycoproteins G-FlpA and G-SodB.

Vaccination with these novel glycoconjugates and their cognate unglycosylated forms resulted in the induction of antigen-specific serum IgY, as expected, and responses were of comparable magnitude when glycosylated or unglycosylated versions of a protein antigen were delivered. Glycan-specific serum IgY proved difficult to quantify, owing to the challenge of obtaining highly purified *N*-glycan for ELISA. In the absence of this, we used whole *C. jejuni* cells and *N*-glycan decorated outer membrane vesicles from *E. coli* harbouring the *pgl* locus [30] as coating antigens for ELISA, but background reactivity was high and we did not observe differences in serum IgY levels between groups vaccinated with the glycosylated versus unglycosylated antigens (data not shown). Further, minimal cross-reactivity was observed when serum from FlpA and G-FlpA vaccinated chickens was tested against captured SodB and G-SodB and vice versa. Together, these observations suggest a lack of significant anti-glycan responses with the regimen used. Despite the induction of antigen-specific serum IgY, vaccination did not significantly reduce the caecal load of *C. jejuni* as previously observed [47]. While responses to SodB or G-SodB were broadly similar between the minimum dose and high dose challenge studies, they were higher for the FlpA and G-FlpA proteins in the minimum dose challenge experiment. This may reflect the use of separate batches for birds, as we have previously observed between replicated vaccine trials [25].

Other studies have failed to detect a clear association between antigen-specific IgY and protection against *Campylobacter* in chickens; for example Chintoan-Uta et al. reported that the peak in anti-SodB responses was not coincident with the reduction in caecal colonisation in chickens vaccinated with a SodB-glutathione-S-transferase (GST) fusion and, moreover, the bulk of SodB was found to be located in the bacterial periplasm [25]. Chemical bursectomy by the cyclophosphamide treatment of chicks, which primarily affects the B lymphocyte compartment, has been reported to reduce clearance of *C. jejuni* from the jejunum and ileum of birds challenged at 3 weeks of age and sampled up to 7 weeks of age, although no effect was detected in the caecum [47]. In studies of a longer duration, B cells and secretory IgA were associated with control of *C. jejuni* in the caeca by 9 weeks post-infection, although the authors acknowledged the potential for a cyclophosphamide-sensitive non-B cell compartment to be involved. The serum IgY levels detected herein may not reflect sIgA responses at relevant sites of colonisation.

To determine if the challenge dose of *C. jejuni* affected the outcome of vaccination, two independent trials were performed, one with a high challenge dose of 10^7^ CFU of M1, as used previously [21,25], and another with the minimum dose required for reliable colonisation, determined in this study to be 10^2^ CFU of M1. Use of the minimum dose did not reveal protective effects, but it is noteworthy that the caecal load of *C. jejuni* rapidly reached over 9 log_10_ CFU g^−1^, even following the delivery of just one hundred viable bacteria. The levels of natural exposure of broilers to *C. jejuni* in poultry production settings are hard to estimate, but vast numbers can be shed when the caeca empty and transmission as a result of coprophagy is predicted. Studies using contaminated litter or involving indirect challenge via the introduction of ‘seeder’ birds colonised by *C. jejuni* would have merit because they simulate field exposure. Indeed, in a study on the effect of faecal microbiota transplants on *C. jejuni* colonisation in chickens, the protective effect was greater in a seeder-bird challenge model than following oral gavage with *C. jejuni* [48].

The results of this study are in contrast with previously published observations with FlpA- and SodB-based subunit vaccines reported to reduce caecal carriage of *C. jejuni* in chickens [18,25]. This could be due to differences in the design of the vaccination trials, the challenge strain of *C. jejuni*, chicken line, timing of administration, adjuvants or the vaccines themselves. The vaccination schedule selected for this study was identical to that used by Neal-McKinney et al. to evaluate FlpA as a *C. jejuni* vaccine. However, it was previously tested against a different *C. jejuni* strain (F38011) and in broiler chickens, and not against *C. jejuni* M1 in layer chickens, as in this study. Moreover, even though the vaccines in both studies were delivered intramuscularly and using the same adjuvant, Neal-McKinney et al. vaccinated chickens first with a GST-tagged 90 mer peptide followed by a His-tagged full-length FlpA protein. In this study, His-tagged full-length proteins and glycoproteins were used to vaccinate chickens at both time points. Similarly, although full-length SodB was used here, the antigen used by Chintoan-Uta et al. was fused to GST [49], and this could potentially have resulted in a distinct tertiary conformation of SodB compared with the His-tagged variant tested in this study. GST itself has been shown to be immunogenic [49,50], which could have contributed to the efficacy of SodB in the previous study. The differences in vaccination schedules between the two studies may also have affected their outcomes. Chintoan-Uta et al. vaccinated chickens on the day of hatch and on day 14 of life, challenged them with a high dose of *C. jejuni* M1 at day 28, and observed a reduction in caecal colonisation by *C. jejuni* at 28 days post-challenge. Despite sampling birds at 28 days post-challenge in this study, no reduction in caecal colonisation was observed. The regimen from the study by Neal-McKinney et al. was selected in order to determine if protection could be observed within six weeks of life in order to be useful in a commercial context.

The addition of the *C. jejuni N*-glycan to our vaccines also did not provide protection against *C. jejuni* challenge, as previously reported [29]. This could be explained by the differences in vaccination schedules and the composition of the vaccines themselves, despite them being tested in layer chickens in both studies. The *N*-glycan vaccine described in Nothaft et al. was composed of nine consecutive acceptor sequons for the addition of *C. jejuni N*-glycans fused to the C-terminus of a truncated and inactive variant of the *Corynebacterium diphtheriae* toxin ToxC, whereas G-FlpA and G-SodB in this study contain two and three *N*-glycans sequons, respectively. While the addition of *N*-glycans at one or all of the available glycosylation sites in G-FlpA and G-SodB is evident by western blotting, it is possible that our vaccines contained an insufficient amount of *N*-glycans to confer protection owing to the presence of unglycosylated proteins in the preparations. Moreover, ToxC, which is known to be immunogenic via T cell stimulation and also functions as a natural adjuvant, could enhance the protective effects of the *N*-glycan [29]. Thus, it is theoretically possible to increase the efficacy of glycoconjugate vaccines against *C. jejuni* by increasing the number of glycosylation sequons and improving glycosylation efficiency to obtain maximum decoration with *N*-glycans. Vaccine efficacy may also be enhanced by adding *C. jejuni N*-glycans to proteins which are known to be highly immunogenic, such as inactivated *Pseudomonas aeruginosa* exotoxin A (ExoA), as reported previously for glycoconjugate vaccines against *Shigella* and *Francisella* [40,51,52].

While we did not observe protection by the novel glycoproteins generated and tested here or their cognate unglycosylated forms, this could be due to the variability in study design, vaccine design and delivery or the *C. jejuni* challenge strain. Moreover, it is important to note that considerable variation can exist for a candidate *Campylobacter* vaccine tested across multiple independent replicates. For example, while a *S*. Typhimurium *aroA* vaccine vectoring CjaA reduced caecal *C. jejuni* levels by 1.4 log_10_ CFU g^−1^ across six independent biological replicates, in some replicates negligible protection was detected, while in others the reduction in caecal colonisation was closer to 3 log_10_ CFU g^-1^ [21]. Further, these data were themselves inconsistent with the 6 log_10_ reduction for a near-identical vaccine reported previously [20]. In turn, this highlights the need for the adoption of consistent protocols that allow a direct comparison of efficacy across trials and for vaccines to be tested repeatedly and against diverse bacterial strains. With refinement of glycoconjugate vaccines by altering the levels of glycosylation, the protein carrier and the regimen, we contend that an efficacious *C. jejuni* vaccine should be feasible.

## Supplementary Materials

The following are available online at https://www.mdpi.com/2076-393X/8/3/520/s1, Figure S1: Schematic of constructs, Figure S2: Specificity of IgY detection.
